# Revolutionizing clinical skills training with Generative AI: the Intelligent Inquiry Training System framework

**DOI:** 10.3389/fmed.2026.1781314

**Published:** 2026-04-24

**Authors:** Hongtao Liu, Huihui Cheng, Yun Zhang, Ying Huang, Danting Tan, Zheng Wang, Fan Zhang, Shaopeng Ming

**Affiliations:** 1 School of Public Health and Health Management, Guangxi Health Science College, Nanning, China; 2 Department of Anesthesiology, The East Hospital of Renmin Hospital of Wuhan University, Wuhan, China; 3 Nursing Teaching and Research Section, Guangxi Traditional Chinese Medical School, Nanning, China; 4 Department of Anesthesiology, Liuzhou Workers Hospital/The Fourth Affiliated Hospital of Guangxi Medical University, Key Laboratory of Anesthesia and Perioperative Neuroprotection of Liuzhou, Perioperative Neuroprotection Engineering Technology Research Center of Liuzhou, Liuzhou, China

**Keywords:** clinical skills training, generative artificial intelligence, Intelligent Inquiry Training System, medical education, virtual patient simulation

## Abstract

**Background:**

Practical training bridges theoretical knowledge and clinical skills in medical education but faces challenges such as resource scarcity, complex patient–physician relationship, and limited interactivity in traditional systems. Generative artificial intelligence (LLMs) offers innovative solutions for simulating virtual patients and enhancing communication training.

**Method:**

An Intelligent Inquiry Training System (IITS) was developed using Baidu’s Wenxin LLM, featuring dynamic case generation, multimodal examination simulation, and emotional feedback modules. Integrated into a blended surgical curriculum, IITS employed pre-class problem-based learning (PBL) and post-class extended training. System efficacy was evaluated via student/instructor questionnaires.

**Results:**

IITS received software copyright certification and demonstrated cross-platform compatibility. Student feedback highlighted its auxiliary role, prompt responses, and need for improved diagnostic accuracy. Teachers praised its case generation efficiency and recommended AI-powered evaluation.

**Conclusion:**

IITS enhances clinical reasoning and communication skills through realistic virtual training, reducing teacher workload. Future iterations will integrate AI scoring and simulator mannequins to validate long-term impacts on clinical competency.

## Introduction

In the field of medical education, practical training serves as a crucial bridge connecting theoretical knowledge with clinical skills, playing a pivotal role in the development of medical students into competent doctors with practical capabilities (1). The core of medical education lies in cultivating medical students’ “practical wisdom,” which encompasses the practical application of theoretical knowledge and clinical decision-making abilities, with practical training being the central pathway for nurturing these competencies. Through early exposure to clinical practice, medical students not only deepen their understanding of disease pathophysiology but also hone their diagnostic acumen and communication skills (2). These soft skills are essential for establishing positive patient–physician relationship, enhancing patient satisfaction, and improving treatment outcomes (3).

However, current practical training for medical students faces multiple challenges. On the one hand, in the context of China’s medical education expansion, the number of medical students has increased significantly, while the growth of high-quality clinical teaching resources has remained relatively inadequate, resulting in a persistent shortage of teaching beds (4). Consequently, medical students have fewer opportunities for hands-on practice in clinical settings, limiting their exposure to a diverse range of diseases and hindering the development of their clinical skills. On the other hand, the current doctor-patient relationship is complex, with some patients exhibiting distrust towards medical students and being unwilling to undergo diagnostic or therapeutic procedures performed by them. This situation imposes considerable psychological pressure on medical students. Additionally, in the process of medical student cultivation, the theoretical learning phase often places emphasis on the imparting of professional knowledge while relatively neglecting the specialized training in effective communication skills with patients. The absence of such training results in medical students generally lacking essential communication skills upon entering the internship stage, making it difficult for them to keenly perceive and attach importance to patients’ emotional feelings. Due to their inability to establish an effective communication bridge with patients, medical students encounter numerous difficulties in obtaining patients’ medical history information, thereby increasing the likelihood of patients developing dissatisfaction and filing complaints (5, 6).

Although traditional computer-based inquiry training systems, serving as auxiliary teaching tools, have to some extent alleviated the issue of scarce teaching resources, they suffer from deficiencies in interactivity and flexibility. The case libraries of existing systems are limited, enabling only mechanical question-and-answer interactions that follow pre-set procedures. Consequently, these systems struggle to adapt to the complex, diverse, and ever-changing disease conditions encountered in clinical practice. Medical scenarios in the real world are fraught with uncertainties, where patients present with a wide range of symptoms that may overlap or vary significantly (7). Due to their inability to break free from pre-defined frameworks, traditional systems find it challenging to provide diagnostic recommendations or treatment plans that are tailored to individual differences and adaptable to changing circumstances, resulting in a notable gap between their capabilities and the actual demands of clinical practice.

In recent years, the rapid development of generative artificial intelligence large language models (LLMs), exemplified by ChatGPT, has offered novel solutions for medical education (8). LLMs have demonstrated significant advantages in medical education, particularly in the simulation of virtual patients and the training of doctor-patient communication skills (9). For instance, researchers at the University of California, Los Angeles, utilized ChatGPT-4 to develop virtual patients, enabling medical students to rehearse discussions with patients regarding abnormal mammogram results. The findings revealed a high degree of realism in the virtual patients, who were capable of exhibiting emotions, thereby facilitating more dynamic and humane interactions between learners and virtual patients (3). These studies suggest that LLMs can accurately replicate disease scenarios, providing medical students with repeated opportunities to practice history-taking and physical examinations, ultimately enhancing their clinical thinking and decision-making abilities.

In view of this, our team has developed an Intelligent Inquiry Training System (IITS) agent based on Baidu’s Wenxin Large Language Model. The IITS is designed to tackle the issues of inadequate interactivity and flexibility in traditional inquiry training systems by intelligently generating dynamic medical cases and simulating real-world clinical inquiry environments. Its objective is to foster the enhancement of medical students’ clinical thinking abilities and communication skills. In this study, we integrated the IITS into a blended teaching approach within medical students’ clinical courses. We constructed a teaching model that incorporates theoretical instruction, interactive inquiry sessions, problem-based learning (PBL) guidance, integration of medical humanities, and post-class reinforcement. The effectiveness of the system was evaluated through feedback collected from both students and teachers.

## Methods

### Software design

The IITS agent, developed based on Baidu AppBuilder,^1^ is dedicated to constructing an efficient, intelligent, and highly clinically relevant teaching environment. The system primarily comprises three core modules: the User Interaction Layer, the Intelligent Clinical Inquiry Engine Layer, and the Teaching Management and Evaluation Layer.

Among these, the User Interaction Layer serves as the system’s frontend, offering an intuitive and user-friendly interface that supports natural language dialogue between medical students and virtual patients. By simulating language communication in clinical consultation scenarios and reproducing the process of virtual examinations, an immersive learning context has been created for students.

The Intelligent Clinical Inquiry Engine Layer, as the system’s core component, operates on two fronts: Firstly, it leverages the generative capabilities of Baidu’s ERNIE 4.0 (Enhanced Representation through kNowledge IntEgration 4.0) large language model to generate real-time patient responses, physical examination findings, laboratory test results, and imaging reports based on students’ inquiry inputs. Secondly, it incorporates advanced natural language processing capabilities to accurately parse complex information within physician-patient dialogues and provide medically logical responses. Developed through customization on the Baidu AppBuilder platform, this layer integrates prompt templates constructed from years of clinical practice and teaching expertise. Through these prompts, the system dynamically simulates diverse clinical scenarios. Educators can effortlessly modify preset disease names, enabling the system to generate complete inquiry content and contextual simulations corresponding to the specified disease in real-time during student-system interactions.

### Instructional application

This study integrated the Intelligent Interactive Training System (IITS) into the Surgery course for Undergraduates Majoring in Clinical Medicine in 2023 (*n* = 700) through a dual-phase instructional approach:

#### In-class PBL-based application

Adopting a problem-based learning (PBL) framework, we structured each theoretical session around specific disease modules. Following the didactic presentation of pathological mechanisms and diagnostic principles, students engaged in simulated clinical consultations via the IITS platform. This allowed real-time practice of history-taking skills while receiving automated feedback on communication techniques and diagnostic reasoning. Teachers subsequently conducted targeted debriefing sessions to address knowledge gaps and procedural misconceptions identified during the simulations.

#### Post-class extended training

To reinforce learning outcomes, we implemented a blended learning model by assigning IITS-based homework assignments. These included:

1. Conducting structured virtual patient encounter training via preconfigured system links.
2. Electronic medical record (EMR) documentation exercises requiring synthesis of acquired clinical data.

Finally, we employed structured questionnaires to quantitatively assess the experiences of both students and teachers regarding the Intelligent Interactive Training System (IITS). The student questionnaire comprised three dimensions:

1. Usage Behavior Characteristics

- Frequency of use (daily/3–5 times weekly/1–2 times weekly/monthly).
- Duration per session (≤15 min/16–30 min/31–60 min/>60 min).
- Usage contexts (in-class practice/after-class autonomous training/pre-exam review).
  2. System Efficacy Evaluation
- Measured via 5-point Likert scales for response timeliness (“extremely prompt” to “severe delay”), content accuracy (“completely accurate” to “frequently erroneous”), and resource adequacy (“highly sufficient” to “extremely inadequate”).
  3. Interaction Experience Assessment
- Solution quality satisfaction (4-point scale: very satisfied/satisfied/neutral/dissatisfied).
- Interface usability measured by 7-point semantic differential scales (1 = extremely difficult to use, 7 = extremely easy to use).
- Open-ended questions collecting improvement suggestions (e.g., “enhancing diagnostic logic”).
- Multiple-choice questions on access preferences (desktop web/mobile app/tablet client).

The instructor questionnaire focused on pedagogical applicability, including:

1. Professional judgment on whether AI virtual patients comply with Clinical Practice Guidelines case design standards.
2. Open-ended inquiries about the system’s educational value and specific areas of assistance (e.g., “history-taking training”).

All student surveys were conducted via electronic questionnaires, while teacher surveys were administered through face-to-face interviews. The electronic questionnaire and its subsequent statistical analysis were both conducted through the Chaoxing Learning Pass platform.^2^ This platform was selected for its reliability and user - friendly interface, which facilitated the efficient collection and processing of data for our research.

This study was approved by Guangxi Health Science College, with the approval number: GXWZY2025109. All participants have been fully informed and have provided their voluntary consent to participate in this research study. This study complies with the Declaration of Helsinki.

## Results

**Figure 1:**
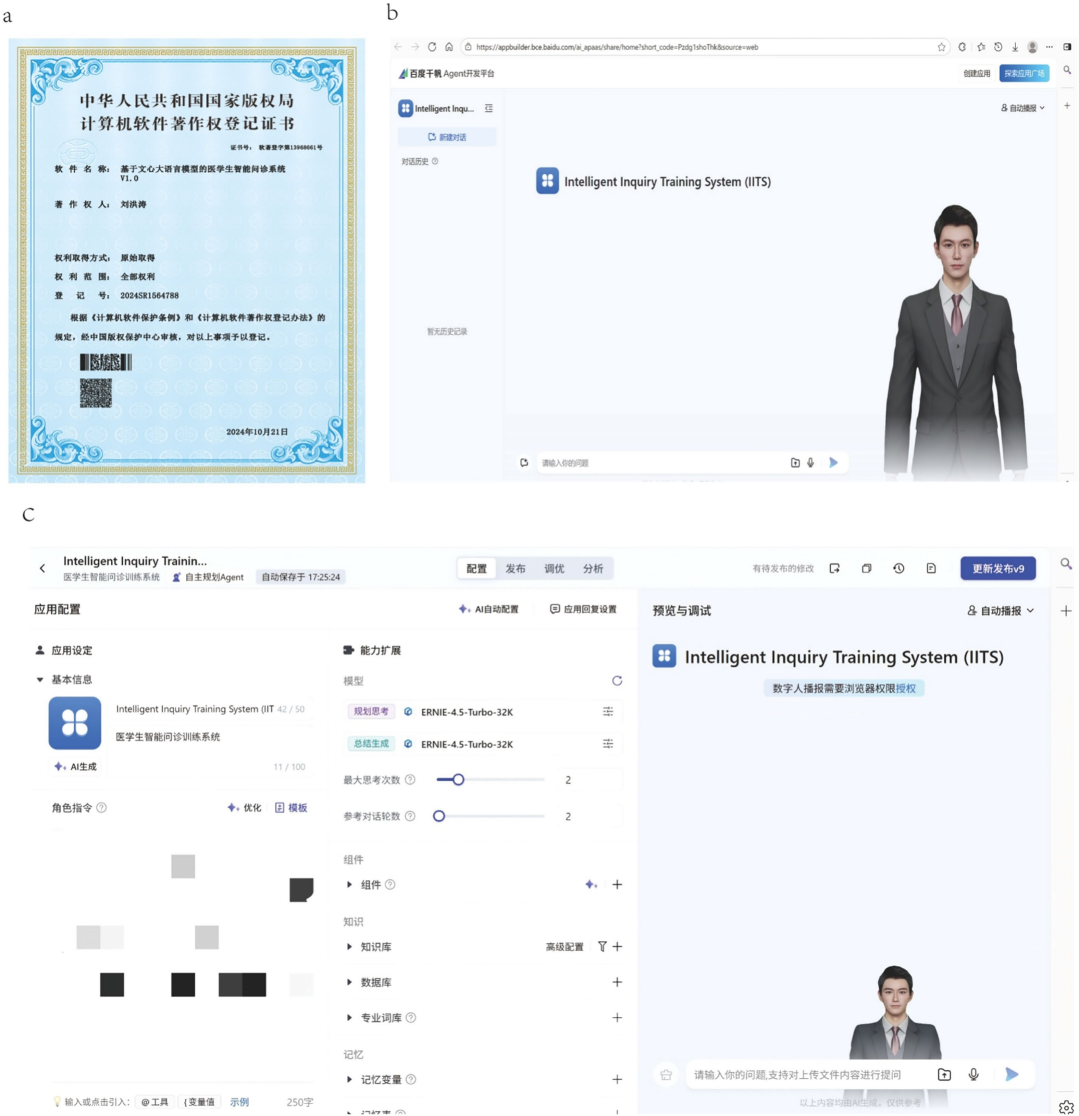
Software copyright certificate and main interface of the software. **(a)** Software copyright certificate; **(b)** Main software interface; **(c)** System backstage.

The IITS, developed via Baidu AppBuilder, received PRC software copyright certification (Reg. No. 2024SR1564788; Figure 1).^3^

The IITS developed in this study incorporates three innovative features:

### Dynamic case generation mechanism

Built upon an advanced large language model architecture, the system constructs personalized diagnostic cases in real time by analyzing student-inputted consultation data around instructor-preset disease names. Employing a database-free design, all case content is dynamically generated through semantic comprehension and contextual reasoning, enabling the production of medically logical yet differentiated outputs for identical consultation inputs. This approach effectively simulates the uncertainty inherent in clinical diagnosis.

### Multimodal examination simulation system

**Figure 2:**
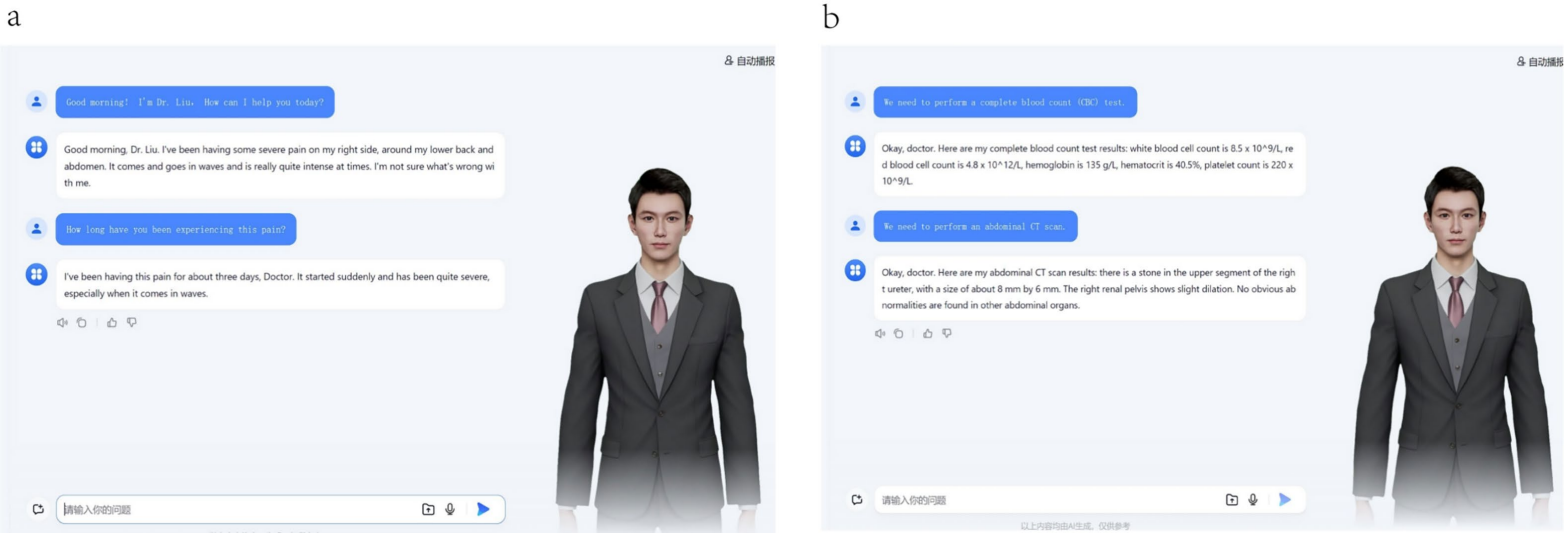
Real-time consultation content generation in IITS. **(a)** Accurate response to student inquiries. **(b)** Dynamic generation of examination results.

The system features a highly adaptable examination module capable of generating physical examinations, laboratory tests, and medical imaging reports (Figure 2) that align with preset disease characteristics. By integrating a digital human interaction interface with dual-channel (voice/text) input support, operational immersion is significantly enhanced.

### Emotional interactive feedback system

**Figure 3:**
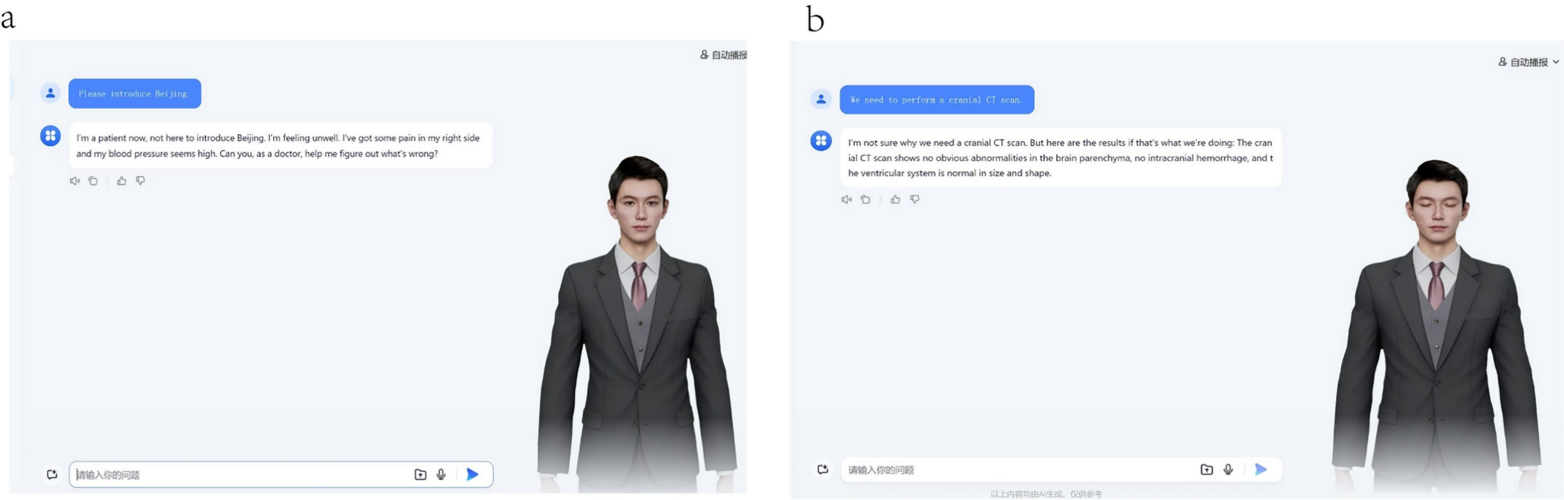
Patient Emotion Configuration. **(a)** The state of urgent anxiety exhibited by the patient during the consultation. **(b)** When patients have objections to the diagnosis and treatment, they will raise questions.

An innovative patient emotional response model has been incorporated, where natural language processing generates affective feedback containing objections when detecting cognitive conflicts between diagnostic recommendations and patient perceptions. For irrelevant consultation content, the system triggers anger emotions consistent with clinical scenarios. This design enables more authentic reproduction of emotional dynamics in physician-patient communication (Figure 3).

### System architecture and cross-platform compatibility

**Figure 4:**
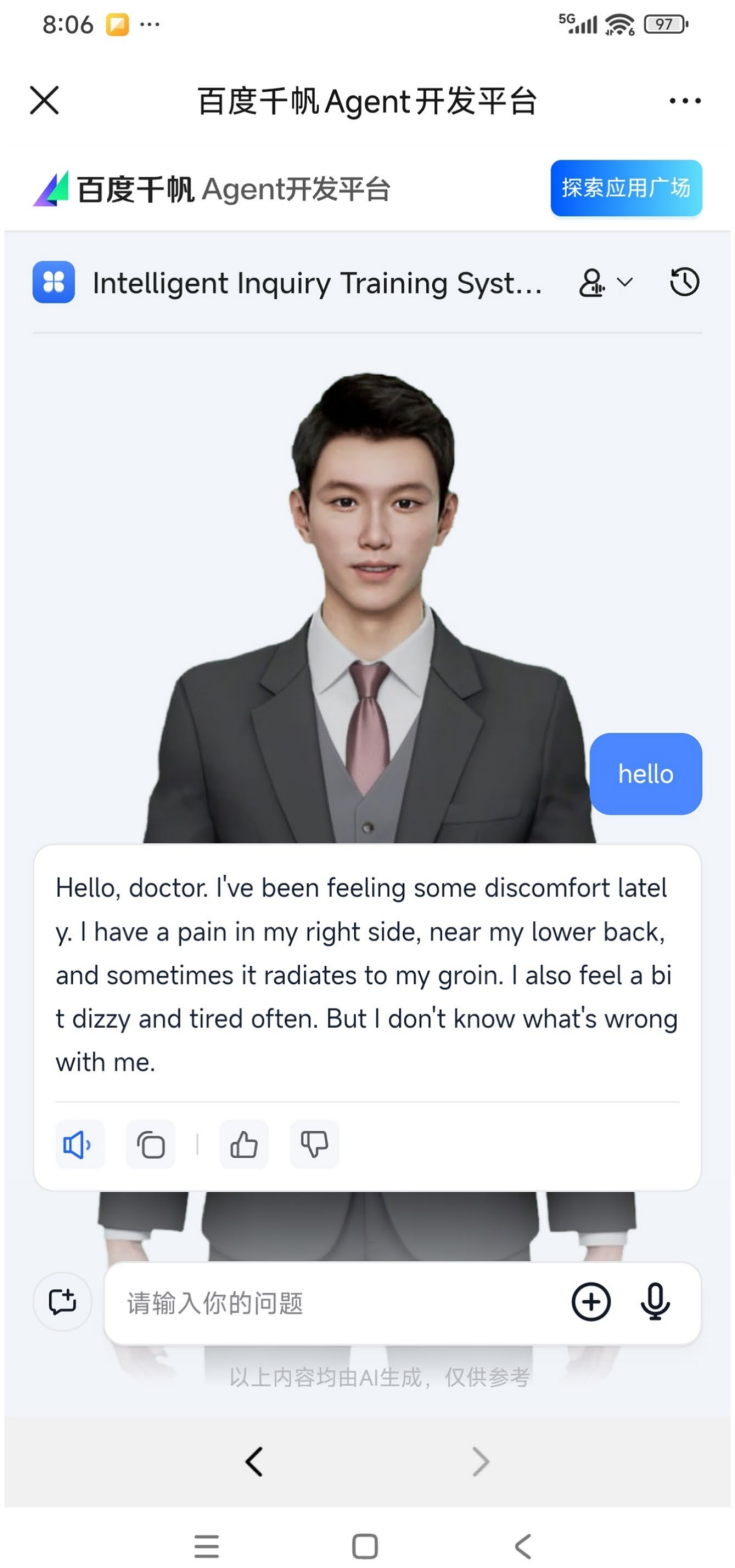
Utilization of IITS on Android mobile platform.

The system employs a web-based modular design architecture, achieving universal platform accessibility through responsive front-end development and standardized protocol interfaces. This design eliminates the dependency on specific hardware environments characteristic of traditional diagnostic simulation systems, enabling seamless transition between classroom PC terminals (Windows/Linux) and mobile devices (Android/iOS/macOS) (Figure 4). Empirical validation demonstrates that the system maintains adaptive interface layouts and functional integrity across diverse operating systems and screen resolutions, with measured touch-interaction response latency below 200 ms on mobile platforms, satisfying the real-time requirements of clinical skills training.

These features collectively create a highly realistic virtual diagnostic environment. The dynamic case generation mechanism overcomes the static limitations of traditional case libraries, the multimodal examination simulation enhances diagnostic thinking training integrity, and the emotional interactive system strengthens physician-patient communication skill development.

**Figure 5:**
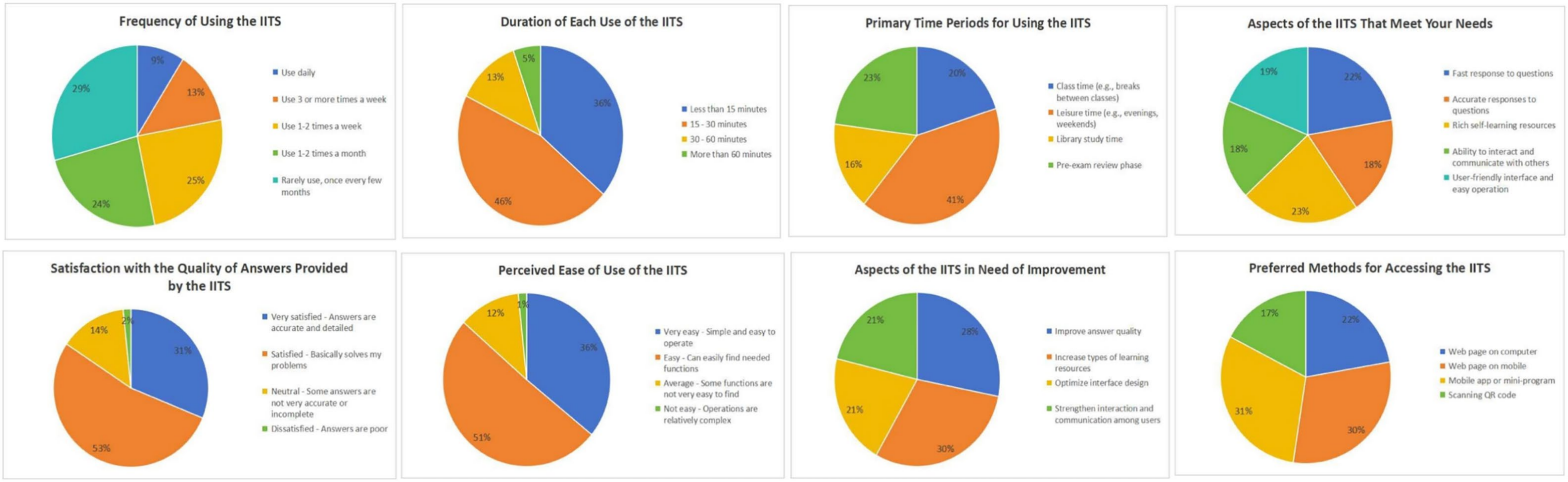
Results of the student questionnaire survey.

We distributed a total of 700 questionnaires and received 487 valid responses. The survey results (as shown in Table 1 and Figure 5) indicate that students’ usage of the “Intelligent Consultation System” exhibits diverse characteristics. In terms of frequency and duration, most students choose to utilize the system during their leisure time, with single-session durations generally kept under 30 min, demonstrating the system’s auxiliary role in students’ daily academic lives. Regarding functionality, students provided high ratings for the system’s question-answering speed and the autonomous learning resources it offers, considering these features to be outstanding. However, students also put forward numerous suggestions for improvement concerning the accuracy of question resolution, user-to-user interaction, and the convenience of interface design. In terms of access methods, students clearly show a preference for using mobile apps, mini-programs, and mobile web pages, reflecting modern students’ reliance on and preference for mobile devices.

**Table 1:** Details of the student questionnaire results.

Serial no.	Question	Option	Number of responses	Proportion
3	After being taught by the teacher on how to use the “Intelligent Inquiry Training System (IITS),” how often do you use it?	A. Daily	44	9.0%
		B. More than 3 times a week	64	13.1%
		C. 1–2 times a week	120	24.6%
		D. 1–2 times a month	117	24.0%
		E. Rarely, a few times every few months	140	29.3%
4	How long do you typically use the “Intelligent Inquiry Training System (IITS)” each time?	A. Less than 15 min	179	36.8%
		B. 15–30 min	220	45.6%
		C. 30–60 min	60	12.3%
		D. More than 60 min	26	5.3%
5	What are the main time periods when you use the “Intelligent Inquiry Training System (IITS)”?	A. Class time (breaks, etc.)	183	37.6%
		B. Spare time (e.g., evenings, weekends)	372	76.4%
		C. When studying in the library	146	30%
		D. During exam review periods	209	42.9%
6	In which aspects do you think the “Intelligent Inquiry Training System (IITS)” meets your needs?	A. Fast response to questions	361	74.1%
		B. Accurate response to questions	294	60.4%
		C. Rich self-learning resources	366	75.2%
		D. Ability to interact with different people	297	61%
		E. User-friendly interface design and easy operation	304	62.4%
7	Are you satisfied with the quality of the answers provided by the “Intelligent Inquiry Training System (IITS)”?	A. Very satisfied, answers are accurate and detailed	152	31.2%
		B. Satisfied, answers basically solve my problems	257	53.2%
		C. Average, some answers are not accurate or complete	68	14%
		D. Dissatisfied, answers are poor	8	1.6%
8	Do you think the interface design of the “Intelligent Inquiry Training System (IITS)” is convenient to use?	A. Very convenient, easy to understand and operate	175	35.9%
		B. Convenient, can easily find required functions	244	50.6%
		C. Average, some functions are not easy to find	58	11.9%
		D. Not convenient, operation is complex	8	1.6%
9	For the “Intelligent Inquiry Training System (IITS),” what aspects do you think need improvement?	A. Improve answer quality	351	72.1%
		B. Increase types of learning resources	375	77%
		C. Optimize interface design	257	52.8%
		D. Strengthen interaction between users	264	54.2%
10	Which ways do you prefer to browse and access the “Intelligent Inquiry Training System (IITS)”?	A. Computer web page	226	46.4%
		B. Mobile web page	307	63%
		C. Mobile APP or mini-program	309	63.4%
		D. Scan QR code	176	36.1%

Through in-depth interviews conducted with four teachers engaged in surgical teaching, we found that all the teachers believed that the virtual patient cases constructed by the IITS align with the characteristics of actual diseases, effectively reducing the workload on teachers in preparing cases before class. Furthermore, the utilization of the IITS facilitates easier identification of issues and deficiencies exposed by students during the learning process. Finally, the teachers strongly recommended the incorporation of an AI evaluation system into IITS.

## Discussion

The reason why our independently developed IITS can authentically simulate a clinical consultation environment is that its core incorporates advanced large language model technology, enabling the system to provide reasonable and context-appropriate responses to a wide variety of students’ questions. In contrast, previous non-intelligent inquiry systems predominantly relied on preset options for questioning, severely limiting students’ freedom in answering and thereby hindering the effective training of communication skills between medical students and patients. Leveraging its highly realistic interactive features, IITS can better train medical students in their communication skills with patients.

Furthermore, IITS possesses the capability to generate examination results of any kind, including those unrelated to the disease. This functionality significantly broadens students’ cognitive horizons and stimulates their thinking. In contrast, traditional inquiry systems typically confine examination items to a preset range within the system, which, to a certain extent, restricts the development of students’ clinical thinking. In real-world clinical practice, physicians enjoy a high degree of autonomy in determining which examinations to order. However, traditional training systems evidently fail to meet this practical need, thereby hindering the cultivation of students’ clinical thinking and decision-making abilities.

Standardized diagnosis and treatment constitute a crucial component of a physician’s professional competence (10). During our long-term teaching practice, we have observed that some students tend to develop an excessive reliance on laboratory or imaging examinations during their academic years, neglecting fundamental skills such as history taking and physical examination. This often leads to misdiagnosis in clinical practice and wastes medical resources. However, this issue is difficult for teachers to promptly identify and rectify during the procedural learning process of traditional inquiry systems. In contrast, within the highly flexible simulated scenarios of our IITS, teachers can easily detect problems in students’ inquiry processes. For instance, when a virtual patient provides only a brief response and a student promptly orders an imaging examination, teachers can swiftly identify the issue by reviewing the student’s inquiry records and provide targeted guidance accordingly. This embodies the core philosophy of our PBL teaching approach, aiming to enhance students’ clinical thinking and problem-solving abilities through highly realistic clinical scenarios.

Moreover, the high degree of freedom inherent in IITS stands as one of the key factors attracting students to utilize the system. Empirical evidence from the gaming industry demonstrates that electronic games with greater freedom of action, such as Minecraft, The Legend of Zelda: Breath of the Wild, and Grand Theft Auto (GTA) (11), tend to captivate players more effectively. Analogously, within a highly flexible inquiry system like IITS, students experience a sense of autonomy in guiding the diagnostic and therapeutic process, which in turn sparks their curiosity and exploratory drive. This observation aligns with the findings from our survey, which revealed a notably high level of enthusiasm among students for engaging with IITS. The current access volume of mobile devices has comprehensively surpassed that of traditional PC platforms. Leveraging its web-based architecture, the IITS achieves seamless compatibility with mainstream mobile operating systems (e.g., Android, iOS), ensuring smooth operation within China’s mobile-dominated internet environment. This design effectively addresses contextual constraints on students’ post-class autonomous training, significantly enhancing both system utilization frequency and student engagement.

Based on the feedback from teachers, when utilizing IITS for teaching, they merely need to input the disease name before class, and the system can automatically generate corresponding case materials. This functionality significantly reduces the workload associated with preparing cases prior to class, thereby earning unanimous recognition from the surveyed teachers. However, given that all responses within the system are generated in real-time and dynamically, teachers are unable to anticipate the specific content of these responses in advance. Consequently, this places more stringent demands on teachers ‘professional knowledge reserves and their ability to respond adaptively.

Given the high complexity of the competency assessment system for medical students (12), we have refrained from conducting a specific evaluation on whether their clinical abilities have improved after using this system. Instead, we have solely focused on investigating students’ levels of interest in utilizing the system. Based on this, we will continue to track the pass rates of these students in future National Medical Licensing Examinations (NMLE) and standardized residency training assessments, and subsequently conduct a comprehensive and systematic evaluation and analysis based on these outcomes. Furthermore, as a single-center study with limited scope of application, this research has certain limitations. In future work, we will extend its application to more clinical courses and promote it to other medical institutions nationwide.

Currently, we are making every effort to advance the development of IITS V2.0. This upgraded version represents a comprehensive enhancement over its predecessor. Notably, it incorporates a wider array of virtual character avatars to elevate the system’s immersion and interactivity. Additionally, an advanced AI-powered scoring function has been integrated, enabling precise and objective evaluation of students’ performance during diagnostic inquiries. Concurrently, a dedicated teacher management portal has been developed, allowing teachers to monitor students’ diagnostic processes and performance in real-time and dynamically. Looking ahead, we plan to deeply integrate the IITS system into simulator mannequins, establishing a multimodal feedback mechanism. This will provide students with a more authentic and comprehensive training experience in clinical diagnostic inquiries.

## Conclusion

The IITS developed in this study effectively fills the gap in medical education by employing large language models to simulate authentic clinical scenarios. Through dynamic case generation, multimodal examination simulation, and affective feedback, the system trains medical students in clinical reasoning, diagnostic thinking, and communication skills. Its flexibility and interactivity have received positive evaluations from both students and teachers, while also reducing the workload of teachers in case preparation. However, its impact on clinical competence requires long-term validation through standardized assessments.

## Data Availability

The raw data supporting the conclusions of this article will be made available by the authors, without undue reservation.

## Footnotes

1
https://cloud.baidu.com/product-s/qianfan_home

2
http://www.chaoxing.com

3
https://appbuilder.baidu.com/s/SeZsT
